# Methyltransferase‐Directed Labeling of Biomolecules and its Applications

**DOI:** 10.1002/anie.201608625

**Published:** 2017-04-10

**Authors:** Jochem Deen, Charlotte Vranken, Volker Leen, Robert K. Neely, Kris P. F. Janssen, Johan Hofkens

**Affiliations:** ^1^ Laboratory of Nanoscale Biology School of Engineering, EPFL, STI IBI-STI LBEN BM 5134 (Bâtiment BM) Station 17 CH-1015 Lausanne Switzerland; ^2^ Laboratory of Photochemistry and Spectroscopy, Department of Chemistry KU Leuven Celestijnenlaan 200F B-3001 Heverlee Belgium; ^3^ School of Chemistry University of Birmingham Edgbaston Birmingham B15 2TT UK

**Keywords:** *S*-adenosyl methionine, DNA functionalization, methyltransferases, protein modification, transalkylation

## Abstract

Methyltransferases (MTases) form a large family of enzymes that methylate a diverse set of targets, ranging from the three major biopolymers to small molecules. Most of these MTases use the cofactor S‐adenosyl‐l‐Methionine (AdoMet) as a methyl source. In recent years, there have been significant efforts toward the development of AdoMet analogues with the aim of transferring moieties other than simple methyl groups. Two major classes of AdoMet analogues currently exist: doubly‐activated molecules and aziridine based molecules, each of which employs a different approach to achieve transalkylation rather than transmethylation. In this review, we discuss the various strategies for labelling and functionalizing biomolecules using AdoMet‐dependent MTases and AdoMet analogues. We cover the synthetic routes to AdoMet analogues, their stability in biological environments and their application in transalkylation reactions. Finally, some perspectives are presented for the potential use of AdoMet analogues in biology research, (epi)genetics and nanotechnology.

## 1 Introduction

Biological systems are complex and never merely the sum of their basic components. Instead, the intricate interplay between countless numbers of biomolecules gives rise to new, “emergent” properties that could not have been predicted when considering each component individually.^1^ In this respect, life itself is perhaps the most intriguing emergent property of all. Nonetheless, even a tiny change in even a single macromolecule can have profound effects on a living organism. Such is the case in the epigenetic regulation of gene activity, where small, highly specific modifications to DNA, RNA, or interacting proteins will result in significant modification of biological function.

Chemists can utilize the inherent specificity of the enzymatic machinery involved in epigenetic regulation to deliver functional or reporter groups to defined macromolecular targets.

**Scheme 1:**
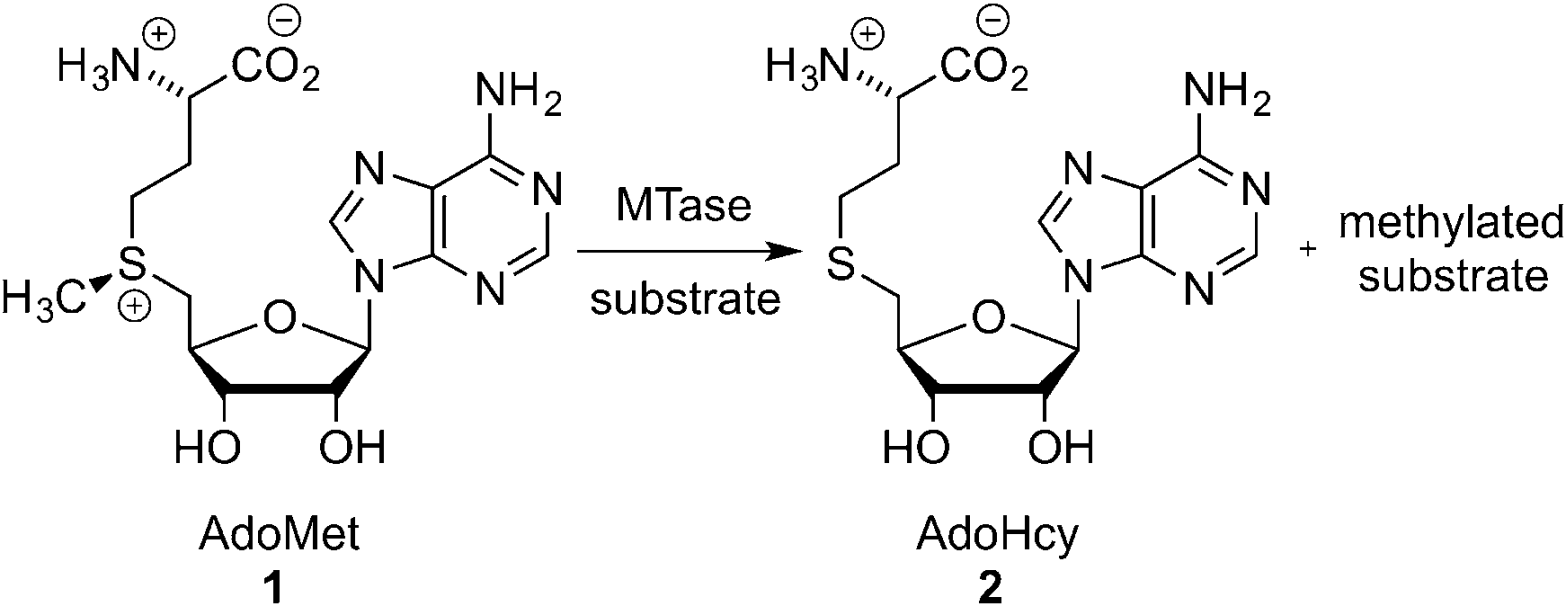
Scheme showing the transfer of a methyl group from the cofactor AdoMet (**1**) to the substrate by the MTase.

This review will focus on a subset of methyltransferase (MTase) enzymes, the *S*‐adenosyl‐l‐methionine (AdoMet **1**, Scheme 1) dependent methyltransferases. In nature, these enzymes methylate their targets, which range from small molecules to proteins and oligonucleotides. They perform this function by binding the methyl donor AdoMet (**1**) and catalyzing the covalent transfer of a methyl group from AdoMet to their target. Moreover, many MTases have been shown to be sufficiently malleable that they will perform a similar catalytic transfer of much more extended and functional moieties than the methyl group. This has allowed them to become an essential tool in the functionalization of the three major biopolymers: RNA, DNA, and proteins. The field has been the focus of increasing attention and over the past decade, several new approaches that allow the incorporation of functional chemical moieties into biomolecules in a site‐specific manner have been developed.

## 2 Labeling Strategies Using AdoMet‐Dependent MTases

AdoMet‐dependent MTases catalyze the transfer of a methyl group from their ubiquitous cofactor to a tremendously diverse range of biomolecules (Scheme 1). This makes them fundamental to a wide variety of biological pathways, including small‐molecule biosynthesis, protein repair, signal transduction, chromatin regulation, and gene silencing.^2^

**Scheme 2:**
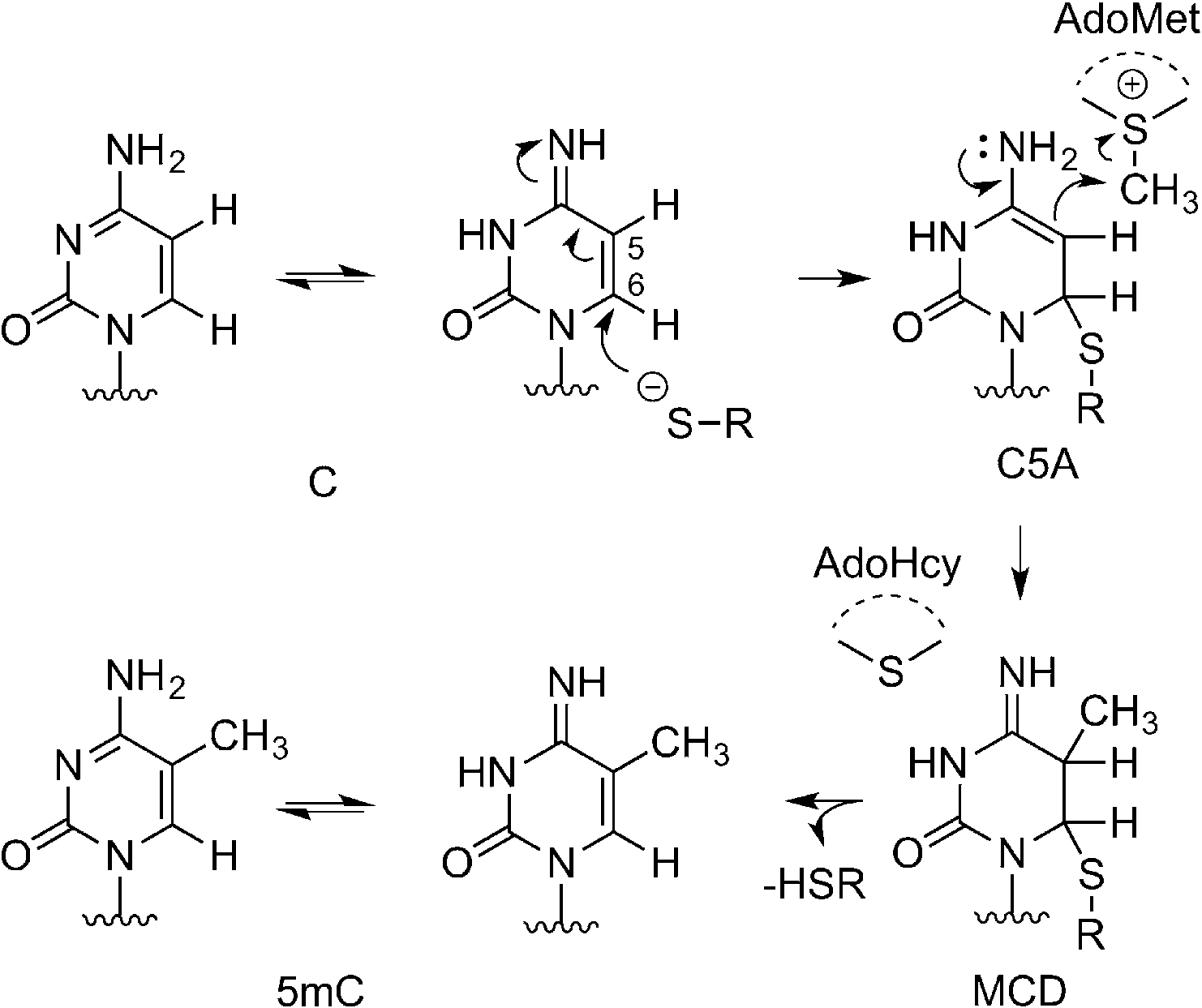
Reaction mechanism of 5‐C methylation by M.*Hha*I

In order to catalyze methylation, the MTases form ternary complexes with their target molecule and AdoMet. These complexes involve a network of intermolecular interactions, but some general principles are common to all systems. The transferable methyl group of AdoMet is bound to a sulfonium center. The molecule is inherently unstable towards nucleophilic attack and the methyl group readily participates in substitution reactions.^3^ This results in a half‐life of 17 hours for AdoMet in the M.*Hha*I reaction buffer at pH 7.4, 37 °C,^4^ and this susceptibility to nucleophilic attack is instrumental to the function of MTases. A mechanism for C5 cytosine methylation by the *Hha*I methyltransferase (M.*Hha*I), which results in 5‐methyl cytosine (5mC), was first described by Santi et al.^5^ The authors proposed a two‐step concerted mechanism (Scheme 2), which is an enzymatically catalyzed version of the Morita‐Baylis–Hillman reaction.5a In the first step of the reaction, cytosine undergoes nucleophilic attack at C6 by the thiol of a cysteine residue in the catalytic pocket of M.*Hha*I. This results in the 6‐Cys‐cytosine compound (C5A). Irreversible nucleophilic attack at the transferable methyl group by C5A results in the stable intermediate 5‐methyl‐6‐Cys‐5,6‐dihydrocytosine (MCD) with simultaneous conversion of AdoMet into AdoHcy.5b Deprotonation and subsequent release of the enzyme of MCD restores the aromatic conjugation and results in the DNA 5‐methylcytosine (5mC) product.5a In N6‐adenine DNA MTases, the 6‐amino group of adenine forms two hydrogen bonds, which increases the electron density of N6 and contributes to its activation for nucleophilic attack.

This mechanism is not specific to M.*Hha*I and has been shown to apply to many other MTases.^3^, ^6^ An S_N_2 mechanism is proposed at the sp^3^ carbon center, with S‐adenosyl homocysteine (SAH) acting as a good and stable leaving group. This has proven instrumental in the development of a large variety of artificial AdoMet analogues.

### 2.1 AdoMet Analogues

AdoMet is a structural hybrid of methionine and adenosine for which both the *R* and the *S* diastereoisomers bind similarly to MTases. However, only the *S* diastereoisomer has the correct geometry at the sulfonium center to allow proper catalytic function.^7^ In nature, the active *S* diastereoisomer is formed through the stereospecific reaction of l‐methionine (l‐Met) with adenosine triphosphate (ATP), which is catalyzed by the methionine adenosyltransferase (MAT) enzymes.^8^

**Scheme 3:**
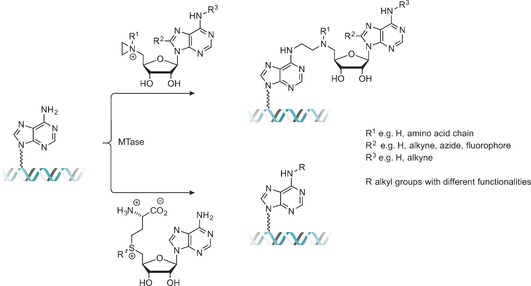
DNA labeling using aziridine‐based (top) or doubly activated AdoMet analogues (bottom).

The vast majority of artificial AdoMet analogues can be categorized into two major groups: aziridinoadenosines and doubly‐activated AdoMet analogues (Scheme 3). To date, the aziridinoadenosines have only been prepared synthetically. However, the doubly‐activated AdoMet analogues can be prepared either through total synthesis or enzymatically, using a synthetic methionine analogue and a MAT enzyme.^9^

#### 2.1.1 Aziridine‐Based AdoMet Analogues

**Scheme 4:**
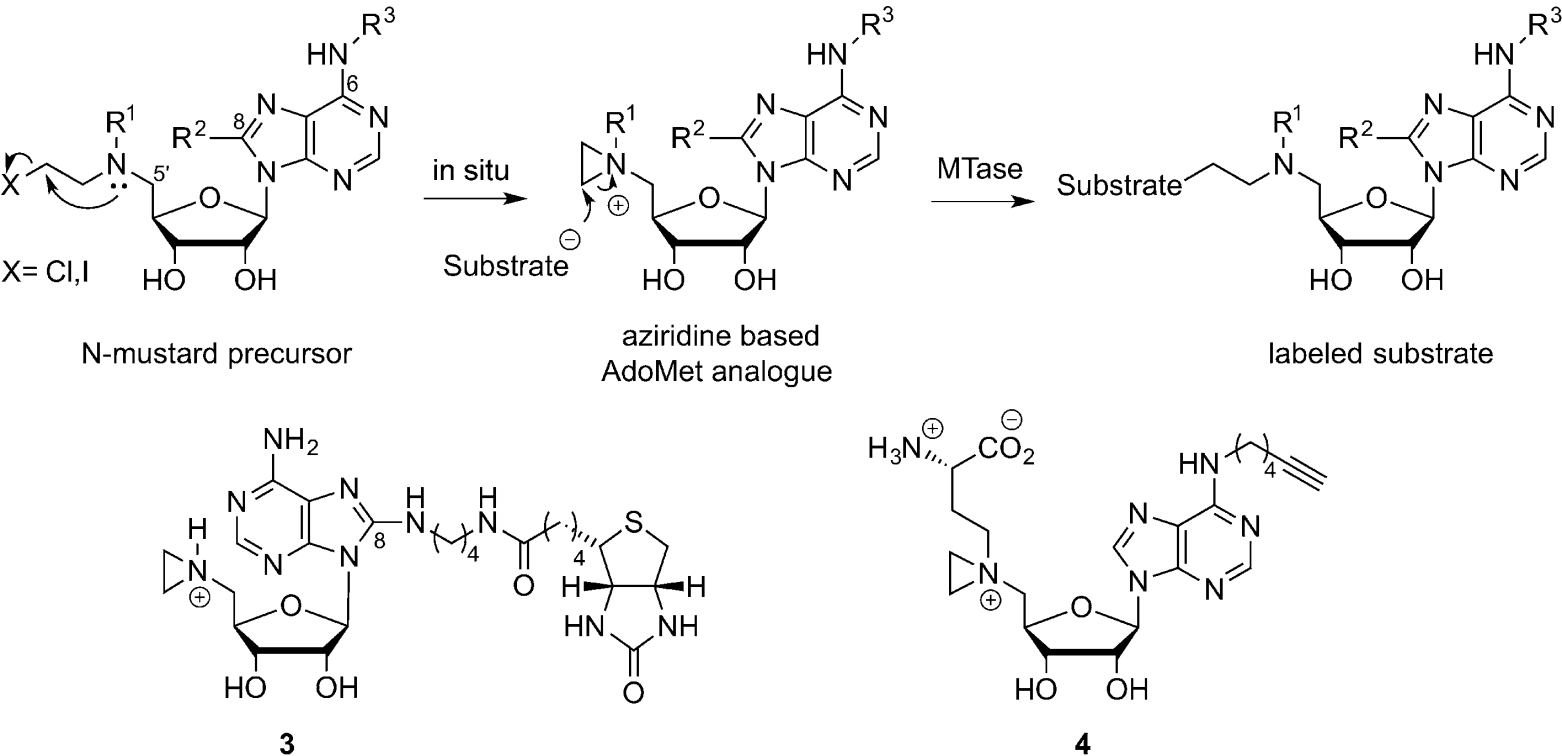
Aziridoadenosine‐based AdoMet analogues.

Aziridine‐based AdoMet analogues (Scheme 4) constitute some of the earliest examples of AdoMet analogues and were first developed by the Weinhold group.^10^ These AdoMet analogues differ from natural AdoMet in that their 5′‐sulfonium is replaced by an aziridine ring (**11**, Scheme 8). This aziridine group undergoes ring opening as a result of nucleophilic attack by the target compound. Since the “leaving group” is conjugated to the transferable moiety in this case, following the S_N_2 reaction mechanism to completion sees the transfer of the entire cofactor analogue to the target molecule (DNA in Scheme 3).^7^

Several DNA MTases were found to be capable of transferring these compounds to DNA. The process of introducing reporter groups (e.g., biotin (**3)**, Scheme 4) to a variety of DNA sequences has been termed “sequence‐specific methyltransferase‐induced labeling” (SMILing). Many different reporters, for example, fluorescent dyes have been attached to the adenine moiety of this aziridine AdoMet analogue for application in DNA labeling.10b, 11 Modelling of the AdoMet binding pocket from crystallographic data showed that steric interactions between the cofactor analogues and the enzyme are likely a key factor determining compatibility of AdoMet analogues with specific methyltransferases. As an example, modelling of the *Taq*I methyltransferase (M.*Taq*I) AdoMet binding pocket showed that the 8‐position of the adenine moiety of a bound cofactor is accessible to the solvent. This implies that the enzyme is therefore likely to tolerate cofactor analogues that incorporate bulky modifications at this site. Successful examples of such bulky modifications to the cofactor analogue include an azide group (**12**, Table S1 in the Supporting Information) and a range of fluorophores.^11^, ^12^ Pljevaljčić et al. identified similar opportunities for modification of the adenine moiety (6‐, 7‐ or 8‐ position) for a range of MTases from their crystallographic structures.^10^

Further work on aziridine‐type AdoMet analogues has focused on the preparation of 5’‐N‐substituted nitrogen mustard (N‐mustard) compounds rather than direct synthesis of the corresponding aziridine. These N‐mustards were found to be reasonably stable and have the ability to form an aziridinium ion in situ (Scheme 4). This approach largely avoids the synthetic difficulties associated with the inherently unstable aziridine moiety.^13^, ^14^ In addition, 5’‐N amino acid substituted N‐mustards (**13**, Table S1) were shown to be significantly more active in MTase directed transalkylation of DNA compared to their counterparts featuring alkyne substituents. Similarly, inclusion of a functional group such as an azide (**14, 15** in Table S1) or a terminal alkyne (**4** in Scheme 4, **16** and **17** in Table S1) enables the use of these N‐mustard cofactor analogues in bioorthogonal ligations.^12^, ^13^

The use of the aziridine‐based cofactor analogues for transalkylation reactions suffers from several drawbacks. Most notably, the transalkylation is not catalytic, and stoichiometric amounts of the MTase must be used in the reaction. This was first observed by Osborne et al., who demonstrated the protein methyltransferase 1 (PRMT1)‐catalyzed transalkylation of a peptide using an N‐mustard‐based cofactor. The product of this reaction, essentially a peptide with a covalently bound cofactor analogue, is a potent inhibitor for the MTase.^15^ Hence, the reaction is self‐limiting. Furthermore, the aziridine‐based analogues are very reactive. This makes them prone to rather rapid degradation and also results in an increased propensity to effect non‐specific alkylation, even in the absence of MTases.^7^

#### 2.1.2 Doubly Activated AdoMet Analogues

**Scheme 5:**
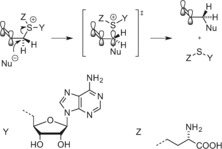
Stabilization of the S_N_2 transition state.17b.

MTases have an inherent ability to catalyze the transfer of alkyl groups larger than methyl groups. However, for DNA MTases, the transfer rate decreases rapidly with increasing size of the transferable moiety (methyl>ethyl>propyl). The reaction occurs through an S_N_2 mechanism with inversion of the configuration at the α‐carbon next to the sulfur atom.^16^ As such, incorporation of an unsaturated bond at the β‐position can potentially stabilize the *p*‐orbital of the α‐carbon formed at the intermediate stage of this substitution reaction. Weinhold, Klimašauskas, and co‐workers were able to demonstrate that an allylic or a propargylic carbon–carbon bond at the β‐position relative to the sulfonium center can restore the reaction rate through conjugative stabilization of the S_N_2 transition state (Scheme 5).^17^

This approach to labeling, using so‐called “doubly‐activated” cofactor analogues, has been termed “methyltransferase‐directed transfer of activated groups” (mTAG).^18^ Unlike aziridine‐based labeling, mTAG is a truly catalytic process and does not require stoichiometric amounts of the MTase.17b Even though multiple turnovers per minute can be achieved, the process is still typically an order of magnitude slower than the equivalent methyl transfer.17b

AdoMet analogues with alkyl, alkenyl (**19**, Table S2), and alkynyl (**20**, Table S2) side chains have been prepared and successfully applied in mTAG.^17^

**Scheme 6:**
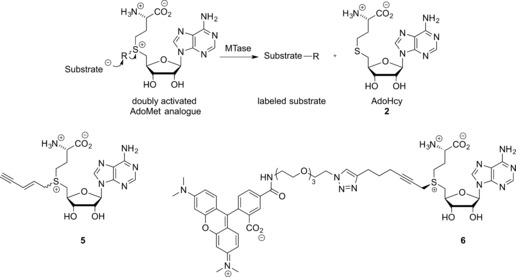
Doubly activated AdoMet analogues.

The approach offers a simple way to label targets with a functional group that can be used for further ligation of more complex moieties. For example, incorporation of different transferrable amine moieties (**21**,^4^, ^18^ **22**,^4^, ^19^ **23**,^4^, 19b Table S2) can be followed by a coupling reaction with the *N*‐hydroxysuccinimide (NHS) ester of an amine‐reactive probe.^18^ In addition, several examples exist on the application of AdoMet analogues with a transferrable terminal alkyne (**5**,^20^ Scheme 6; **20**,20g,20h **24**,20b,20c,20g and **25**,^4^, 19b Table S2) or azide (**26 g**,^20^, ^21^ **27**).^4^ The transferred alkynes and azides can be further functionalized by using the biocompatible and highly‐efficient azide–alkyne cycloaddition reaction (one of the “click” series of reactions). However, some of these analogues, such as compound **20** (Table S2), which features a terminal alkyne, are highly unstable.^4^, 20g,20h Other AdoMet analogues, such as the AdoEnyYn compound (**5**, Scheme 6), are more stable, thus making them more suitable for use in bioorthogonal ligations.20b–20h

Ketones are yet another interesting transferrable moiety since they can be used to react with hydroxylamines and hydrazides. This was demonstrated with AdoMet analogue **28** (Table S2), which was successfully transferred to DNA and subsequently used for coupling with a hydroxylamine coupled to a fluorophore.^22^ Finally, AdoMet analogues directly incorporating a fluorescent dye allow single‐step direct labeling reactions. An example in which a TAMRA dye was coupled to the AdoMet analogue (**6**, Scheme 6) and subsequently used for labeling reactions was first described by Grunwald et al.^23^

### 2.2 Stability of AdoMet Analogues

**Scheme 7:**
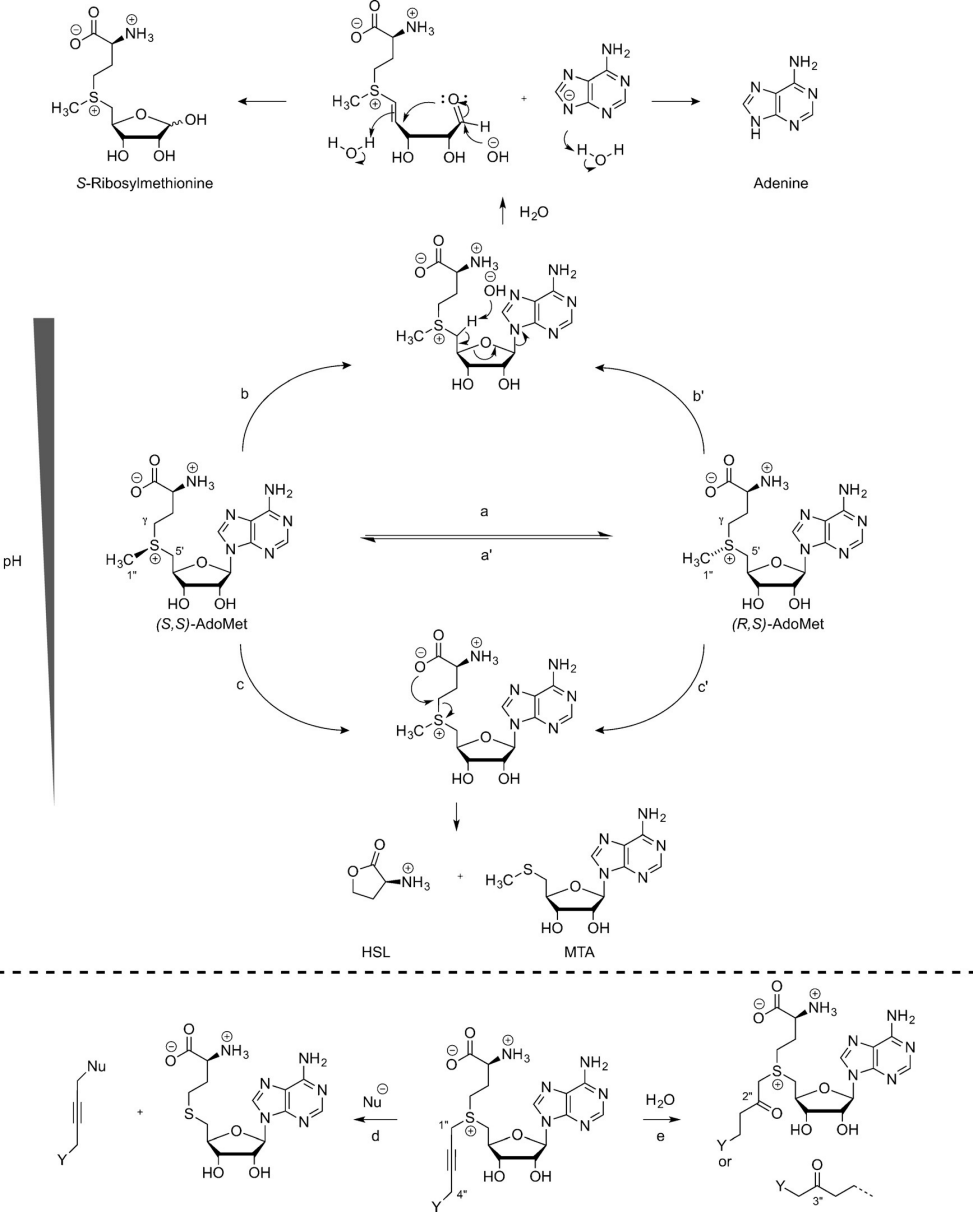
Decomposition pathways of (*S*,*S*)‐AdoMet (and its analogues). a) Inversion at the sulfonium center of *(S*,*S)*‐AdoMet results in (*R*,*S*)‐AdoMet. b) Deprotonation at the C‐5′ and subsequent elimination of adenine base to give *S*‐ribosylmethionine. c) Nucleophilic attack by the α‐carboxylate on the γ‐carbon of methionine delivers HSL and MTA. d) Nucleophilic addition at 1′′‐C. e) Addition of water to the 2′′‐C or 4′′‐C position.

AdoMet analogues are prone to spontaneous decomposition in aqueous environments through a range of different pathways (Scheme 7).^4^, ^24^ Reversible racemization (route a) to the *R* diastereomer of AdoMet is also common and can result in the formation of an inactive AdoMet analogue. The presence of the sulfonium center activates the adjacent carbon atoms towards decomposition reactions. Under alkaline conditions, deprotonation at the 5′‐C occurs (route b), which results in formation of adenine and *S*‐ribosylmethionine. Under more acidic conditions, intramolecular attack by the α‐carboxylate group on the γ‐C of the methionine group (route c) yields methylthioadenosine (MTA) and homoserine lactone (HSL). In the case of AdoMet, no nucleophilic attack is observed at the methyl group, but this changes when considering AdoMet analogues with extended chains containing a triple bond in the β‐position relative to the sulfonium center. The partial positive charge at the 1′′‐C increases, thereby making it more susceptible to nucleophilic attack (route d). When electronegative groups (e.g., Y=NH_2_) are in close proximity to the triple bond, this can lead to a higher electron deficiency at both 4′′‐C and 1′′‐C, which enables the addition of water (route e) to both to give the hydrated compound, which shows almost no reactivity towards most DNA MTases and protein MTases.^4^, ^24^

At pH 7.5 and 37 °C, the rate constants for racemization, cleavage to HSL/MTA, and hydrolysis to adenine/S‐ribosylmethionine were reported to be 1.8×10^−6^ s^−1^, 4.6×10^−6^ s^−1^, and 3×10^−6^ s^−1^, respectively. The hydrolysis rate shows a significant decrease at lower pH values.^25^

Recently, Huber et al. developed novel AdoMet analogues lacking the N7 of the adenine moiety and further omitting the carboxylic acid of the amino acid chain in favor of a tetrazole ring. This makes them less likely to decompose through pathways b and c and restricts epimerization at the sulfonium center.^26^

**Figure 1:**
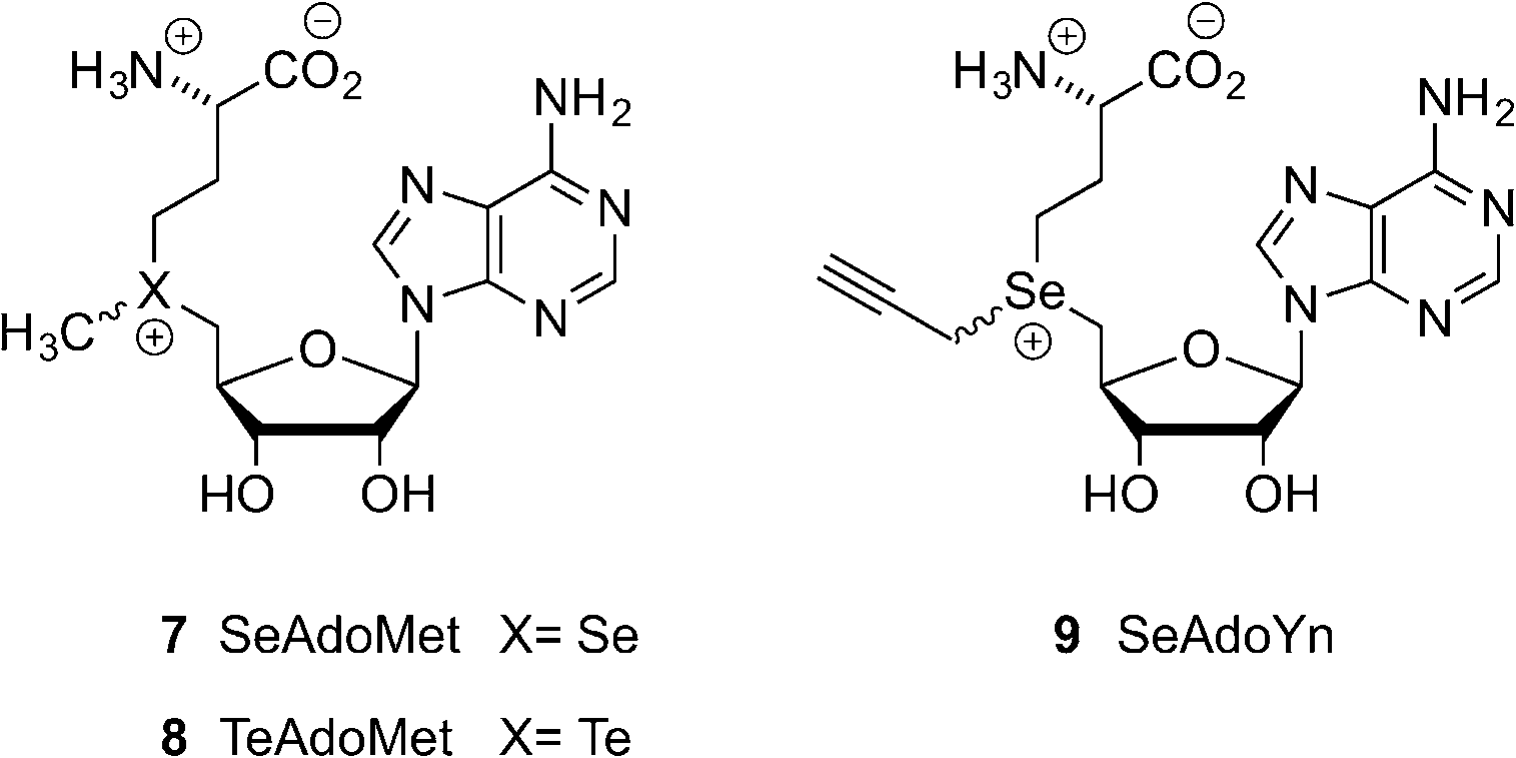
Chalcogen‐containing AdoMet analogues.

To improve the stability and reactivity of the AdoMet analogues, several molecules have been studied in which sulfur is replaced with another chalcogen, such as selenium (SeAdoMet (**7**), Figure 1) or tellurium (TeAdoMet (**8**), Figure 1). For AdoMet, three decomposition pathways are common: racemization to the inactive diastereomer, deprotonation of the 5′‐C to yield S‐ribosylmethionine, and adenine or intramolecular nucleophilic attack by the carboxylate group to give HSL and MTA. SeAdoMet (**7**, Figure 1) was found to decompose via two of these pathways, whereas the tellurium analogue TeAM (**8**, Figure 1) was inert to all three pathways.^24^ It has been shown that the trend in electrophilicity of these AdoMet analogues follows the order SeAdoMet>AdoMet>TeAdoMet and that the 5′‐C acidity follows the order AdoMet>SeAdoMet>TeAdoMet.^27^ Selenium analogues are therefore both more reactive towards nucleophilic attack, thus making them better suited as cofactors for transalkylation, and more stable towards deprotonation of the 5′‐H (pathway b, Scheme 7). As a result, they have been subject of extensive investigation. Weinhold et al. have developed SeAdoYn (**9**, Figure 1) as a cofactor for protein‐methyltransferase‐mediated fluorescent labeling of proteins. This analogue showed improved reactivity and higher stability toward hydrolysis compared to the similar sulfur‐containing AdoEnYn and AdoYn analogues.20h Comparative decay studies with the propargylic SeAdoYn (**9**, Figure 1) and ProSAM (**20**, Table S2) analogues showed that decomposition of the ProSAM compound first results in the hydrated product (keto byproduct **28**, Table S2), which further decomposes to the thioether. However, SeAdoYn follows a different mechanism and directly forms the selenoether.20h, 28 SeAdoYn is used as a substrate for transalkylation by a large variety of wild‐type MTases, which is in contrast with many of the AdoMet analogues with larger transferable groups, which are only active with mutated MTases.9b, 19a, 20h, 28, 29

The Luo group has also demonstrated that selenium substitution can result in increased reactivity compared to the sulfur‐centered AdoMet analogues when applied as substrates for the protein MTases. Their study shows that the decomposition rate of SeAdoMet is 10‐fold higher than that of AdoMet, but the protein‐MTase‐catalyzed transalkylation reaction is only 3–5‐fold faster for the SeAdoMet analogue. Hence, the authors suggest that the protein‐MTase‐catalyzed reaction rates are not determined solely by the strength of the chalcogen–carbon bond, but are likely caused by other factors.^28^, ^30^ Interestingly, the study also suggests that the β‐sp^2^ carbon atom, which is essential for activity with S‐alkyl AdoMet analogues, is likely not required for some protein MTases when Se‐alkyl AdoMet analogues are used.^30^

### 2.3 Synthesis of AdoMet Analogues

**Scheme 8:**
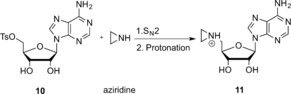
Synthesis of the *N*‐Adenosylaziridine AdoMet analogue. Step 1: Nucleophilic substitution of the tosylate (TsO) group in 5′‐deoxy‐5′‐tosyladenosine with aziridine.

The first example of the synthesis of aziridine‐based AdoMet analogues was described in 1998 by Pignot et al., who focused on the development of aziridine‐based cofactor analogues.10a, 31 *N*‐Adenosylaziridine was synthesized through nucleophilic substitution of the tosylate group in 5′‐deoxy‐5′‐tosyladenosine with aziridine and subsequently activated as an alkylating agent through protonation of the nitrogen atom in the aziridine ring (Scheme 8).10a

The ring strain in this three‐membered heterocycle makes it susceptible to nucleophilic ring opening, which is further facilitated by nitrogen quaternization. This aziridine‐based AdoMet analogue can be used for the introduction of different functional or reporter groups through modification of the adenine moiety. One such example shows the attachment of a dansyl fluorophore at the adenine 8‐position.^11^ The synthesis starts from 8‐bromo‐2′,3′‐*O*‐isoporpylideneadenosine, which was treated with diaminobutane to introduce a flexible linker and was converted in a few steps (substitution and deprotection) into the N‐adenosyl aziridine derivative. A similar route has been used to prepare the N‐mustard precursors of the aziridine‐based AdoMet analogues (Scheme 4).^32^

These aziridine‐based AdoMet analogues suffer from a long and low‐yielding synthetic route.^10^, 12b, 13, 14 Townsend et al. have reported improved synthetic routes for the synthesis of N‐chloromustard‐substituted adenosines using reductive amination as the key step and have also demonstrated the synthesis of a photocaged derivative, which benefits from increased stability compared with standard aziridine‐based cofactors and is readily activated as a cofactor through UV irradiation.^33^

**Scheme 9:**
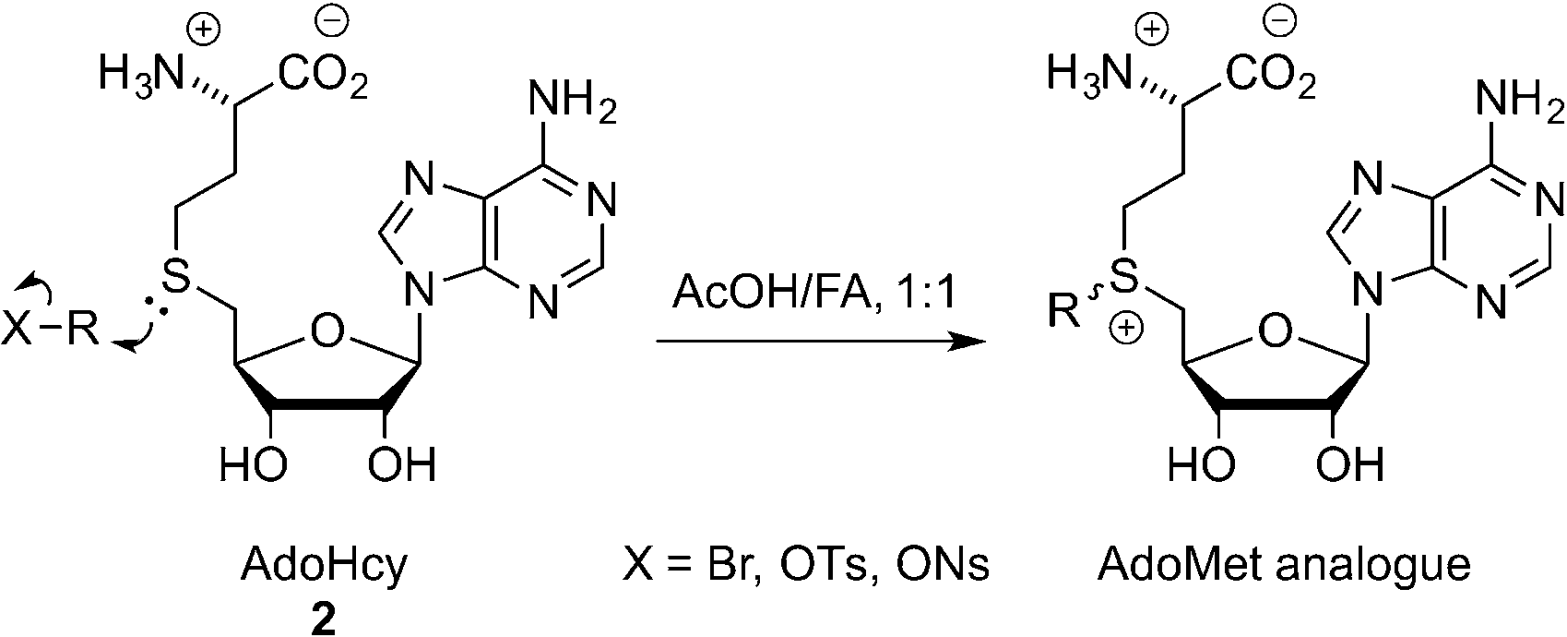
Synthesis of doubly activated AdoMet analogues. FA=formic Acid, Ns=nosyl.

Doubly‐activated AdoMet analogues are typically synthesized by combining *S*‐adenosyl‐l‐homocysteine (AdoHcy (**2**), Scheme 9) with an excess of strong electrophiles, for example, alkyltriflates or alkylbromides, under acidic conditions.^34^ The first chemical synthesis of the diastereomers of AdoMet from AdoHcy was published in 1959.^35^ In an acidic environment, the propensity for nucleophilic attack by AdoHcy amines, hydroxy groups, or the carboxyl acid, for example, is strongly reduced, leaving the thioether as the sole reactant.17a Both the *R* and the *S* epimers are formed during this S_N_2 reaction. However, since only the active *S* epimer can be used in transmethylation reactions, a separation of the diastereomers is preferable. Reversed‐phase HPLC (RP‐HPLC) is commonly used in efforts to separate the diastereomers. This process, however, remains challenging.

The synthetic routes to AdoMet analogues are not trivial. In addition, these compounds often display limited stability. As a result, there have been several attempts have to prepare both AdoMet and its analogues enzymatically.

**Scheme 10:**
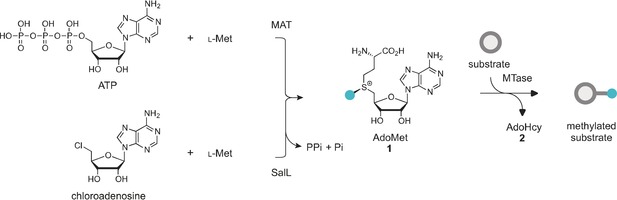
Enzymatic AdoMet synthesis using SalL and MAT.

AdoMet itself can be obtained through isolation from yeast grown in media supplemented with l‐methionine.8b Small‐scale8a, 36 (μmol) enzymatic syntheses of AdoMet from ATP and l‐methionine (l‐Met) as well as larger scale8b (mmol) syntheses have been reported. The AdoMet formation is catalyzed by l‐methionine S‐adenosyl transferase (MAT or AdoMet synthetase) in a two‐step fashion where the complete tripolyphosphate (TPP) chain of ATP is cleaved and subsequently hydrolyzed to pyrophosphate (PPi) and phosphate (Pi). The enzymatic synthesis results in high yields of the preferred epimer (−)‐AdoMet (Scheme 10, top).36a, 37

Recently, several groups have demonstrated enzymatic syntheses of AdoMet analogues as well.^9^, ^38^ The Thorson group tested a range MAT enzymes for their ability to catalyze the formation of several AdoMet analogues. They tested a library of 44 non‐native S/Se l‐Met analogues with five different MATs. Of these, human MAT II was the most permissive. Furthermore, the synthesized analogues could be successfully used in the alkylation of small molecules (indolocarbazoles).9b The Burkart group have demonstrated the chemoenzymatic synthesis of several AdoMet analogues using either a fluorinase (FDAS) from *Streptomyces cattleya* or a chlorinase (SalL) from marine bacterium *Salinaspora tropica*.^38^, ^39^ Both halogenases are known to catalyze the breakdown of AdoMet to give l‐Met and fluoro‐5′‐deoxyadenosine (FDA) or 5′‐chloro‐5′‐deoxyadenosine (ClDA). However, the reaction can be reversed at low chloride/fluoride and high l‐Met concentrations (Scheme 10, bottom). This enzymatic pathway can also be exploited by using various l‐Met derivatives for the enzymatic synthesis of AdoMet analogues.9a, 38, 40 However, it should be noted that decreased activity of the l‐Met analogues with increasing size was observed.^40^

### 2.4 Methyltransferases

Methyltransferases are typically categorized into five distinct families (classes I–V) based on structural similarities.2a Of these, Class I MTases are by far the largest group. They catalyze the majority of methylation reactions and include all DNA MTases and some protein MTases and RNA MTases.2a The largest group of protein MTases are the protein lysine MTases, and together with the protein arginine MTases, they play an important role in histone modification and thus ultimately in gene transcription. Most of the work in the past decade using modified AdoMet analogues has focused on class I MTases and class V MTases, which are also the focus of this Review. Other, less common, classes include the MetH reactivation domain MTases (class II), precorrin‐4 MTases (class III), and the SPOUT family of RNA MTases (class IV).^2^

While the MTases share little sequence similarity, they do share a highly conserved structural fold. The core element of this conserved fold consists of seven‐stranded β‐sheets with three helices on each side, a structural feature commonly referred to as the Rossman fold.2b, 41 This core is shared among MTases that act on all different substrates, ranging from small molecules to DNA and proteins.^3^, ^42^ This structural similarity suggests that the labeling reactions with AdoMet analogues are likely universal and applicable across the entire range of MTases.

In labeling reactions, wildtype MTases are most frequently used in combination with an AdoMet analogue. However, in some cases, this is not possible because the native fold of the MTases is incompatible with the AdoMet analogue. This could be due to bulky groups on the AdoMet analogues that prevent a good steric fit or disruption of the needed binding interaction to the AdoMet binding pocket. In these scenarios, it could prove useful to engineer the AdoMet binding pocket for a more favorable interaction with the AdoMet analogues, for example by directed evolution of the MTase.^43^

In other cases, it might be preferable to use an MTase that interacts more readily with the AdoMet analogue rather than the natural AdoMet, for example, when using the labeling reaction in live cells in the presence of natural AdoMet. In this scenario, it would be useful to engineer the AdoMet binding pocket such that the enzyme prefers the AdoMet analogue over the natural AdoMet. To achieve this, one possibility would be to apply a “bump‐and‐hole” strategy, where point mutations are introduced at the protein active site. Subsequent screening of these mutants can then reveal those variants which display enhanced selectivity towards the AdoMet analogue rather than Adomet.^44^

#### 2.4.1 DNA Transalkylation

In bacteria, DNA MTases play a role in defense of the bacterium against viral invasion by enabling a distinction to be made between the host genome and invading viral DNA.^6^, ^45^ In eukaryotic cells, the main role of DNA MTases is the regulation of genes.^6^ DNA methyltransferases can be subdivided depending on their target for modification (cytosine C5, cytosine N4, or adenine N6). Of these groups, the cytosine C5 and adenine N6 MTases are found in many species of fungi, bacteria, and protozoa, while the cytosine N4 MTases occur only in bacteria.^6^, ^46^

**Figure 2:**
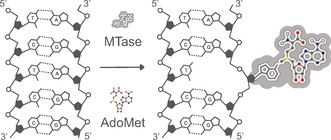
The base‐flipping mechanism of MTase‐mediated DNA methylation. Most of the work on DNA MTases with AdoMet analogues is based on M.*Taq*I, a DNA MTase from the thermophilic bacterium *Thermus aquaticus*.

The DNA MTases typically recognize short, palindromic DNA sequences (2–8 bases long). Ordinarily, the target base for methylation lies buried within the DNA helix and as a result, the DNA MTases extrude this base from the DNA duplex, flipping it into the enzyme catalytic pocket, where transmetylation occurs (Figure 2).^47^ The byproduct of this catalysis, *S*‐adenosyl‐l‐homocysteine (AdoHcy (**2**), Scheme 9), is subsequently released from the AdoMet binding pocket and, in cells, digested by an AdoHcy hydrolase enzyme.^48^

DNA MTases were the first MTases that were shown to catalyze transalkylation reactions using AdoMet analogues. Part of the reason for this is likely the ease with which the progress of the labeling reaction can be followed. Every bacterial MTase has a partner restriction endonuclease that cleaves DNA at the same site targeted by the MTase. However, if that site is methylated (or alkylated), cleavage is prevented. This can be exploited to readily confirm the activity of the methyltransferase with the AdoMet analogue using gel electrophoresis.

The first example of a transalkyation reaction using an AdoMet analogue and a DNA MTase was the aziridine transfer with the M*.Taq*I DNA MTase in 1998.10a Many aziridine analogues have subsequently been successfully transferred to DNA, for example, by the adenine DNA MTases M.*Eco*RI12b and M.*Bsec*I^49^ and cytosine DNA MTases such as M.*Hha*I12a and M.*Sss*I.14a In these cases, functional moieties (including fluorophores)^11^ were placed at various locations of the aziridine cofactor. The aforementioned study by Pljevaljčić et al., which models the cofactor binding pockets of several DNA MTases together with the cofactor analogues, concludes that 4 of the 6 screened enzymes (M.*Taq*I, M.*Rsr*I, M.*Dpn*M, M.*Pvu*II) are likely to tolerate cofactors carrying bulky modifications at the 8‐position of the cofactor adenine moiety. For M.*Hha*I and M.*Mbo*II, the 7 or 6 positions of that same adenine moiety appear to be feasible sites for the attachment of bulky modifications.10b However, to the best of our knowledge, only the M.*Taq*I and M.*Hha*I enzymes have been shown to transfer an aziridine AdoMet analogue to DNA. Unpublished work from our laboratory failed to show any transfer with the M.*Pvu*II enzyme and aziridine analogues carrying modifications at the adenine 6‐, 7‐, and 8‐positions.

As mentioned in Section 2.1.1, the aziridine AdoMet analogues suffer from some disadvantages, which has inspired Weinhold, Klimašauskas, and co‐workers to develop the doubly‐activated AdoMet analogues. These cofactors have been employed in successful transalkylation reactions with several DNA MTases, with either adenine or cytosine as the target base. A comprehensive overview can be found in Table 1. The attachment of a functional or fluorescent group to the DNA can be achieved using a second chemical coupling reaction, such as the coupling of amines to NHS esters19c or click‐chemistry‐based conjugation.20f Alternatively, like the aziridine cofactors, the fluorescent group can be synthetically coupled to the cofactor, thereby allowing one‐step transfer of the fluorescent groups to the DNA substrate. This approach was first demonstrated with the DNA MTase M.*Taq*I.^23^

**Table 1:** DNA MTases shown to be compatible with various AdoMet analogues, and the functionalities implemented on the AdoMet analogue.

			MTAG				Aziridine				
	Target	Alkyl	Alkyne	Amine	Azide	Fluo^[b]^	None	Alkyne	Azide	Biotin	Fluo^[b]^
M.TaqI	TCGA	17	17b, 20f	18		23	10, 11, 12b, 14a	13, 14b	12b, 14b	12b, 49a	10b, 11, 51
M.HhaI^[a]^	GCGC	17b		18, 19c,19d	4		12b	14b	12b, 14b	52	
M.SssI^[a]^	CG			50	50						
M.BseCI	ATCGAT									49	
M.BcnIB^[a]^	CCSGG	17b	17b								
M2.Eco31I^[a]^	GGTCTC		19d	19d							
M.EcoRI	GAATTC						12b, 14a	13	12b		
M.HpaII	CCGG		19d	19d			12b, 14a				
M.XbaI	TCTAGA		20f								
MFokI	GGATG and CATCC		20f								

[a] Mutant enzyme with improved activity for the AdoMet analogue. [b] Fluo=fluorophore.

The rates transalkyation reactions with the doubly‐activated AdoMet analogues have been dramatically improved by engineering of the cofactor binding pocket of the cytosine C5 MTases.19d Three amino acid side chains were selected based on their potential steric interaction with the cofactor, and replaced with shorter residues (two residues were replaced with alanine, while one residue was replaced with the smaller polar residue serine). The mutants showed a significant improvement in both binding affinity and transfer rate with the AdoMet analogues, relative to the wild‐type M.*Hha*I. In general, this effect was more marked for the longer cofactor analogues with transferrable groups with longer alkyl‐chains than those with shorter chains. Furthermore, the mutant enzymes show a significant reduction in methylation rate with the natural cofactor AdoMet. The binding constant of the enzyme with the natural cofactors AdoMet and AdoHcy was reduced as well. The weaker binding of AdoHcy may contribute to the increased catalytic efficiency of the AdoMet analogues.

Two of three mutations were located within the highly conserved sequence motifs of M.*Hha*I and were readily mapped to locations on other C5 DNA MTases. Indeed, the same mutations were successfully applied to the cytosine‐5 MTases M.*Hpa*II and M2.*Eco*31I, and later to the CpG‐specific MTase M.*Sss*I.^50^ However the same mutations did not result in improved labeling when using M.*BsaH*I.20f

#### 2.4.2 RNA Transalkylation

In this section, we present a brief overview of the application of the RNA methyltransferase enzymes that have been shown to catalyze transalkylation of RNA using AdoMet analogues. Early studies have shown some promise in this area but all have been realized in vitro using synthetically prepared or in vitro transcribed RNA substrates. Whether these methyltransferases can replace existing antibody‐based approaches for the targeted labelling or capture of RNA‐based substrates remains an area for further investigation.

The first example of an RNA‐methyltransferase‐mediated transalkylation was with the tRNA methyltransferase Trm1.20d This enzyme has been shown to catalyze the transalkylation of the N2 of guanosine 26 in tRNA^Phe^ using a doubly‐activated SAM analogue with an alkyne functionality. The modified tRNA was subsequently fluorescently labelled using copper‐catalyzed azide–alkyne cycloaddition (CuAAC; one of the “click” reactions) in order to generate fluorescently labeled tRNA.

Using in vitro reconstitution of an archaeal box C/D small ribonucleoprotein RNA 2′‐*O*‐methyltransferase (C/D RNP) Tomkuvienė et al. were able to demonstrate transalkylation of in vitro transcripts of tRNA and pre‐mRNA molecules. The C/D RNP complex includes a guide RNA molecule that is used to direct the specificity of the C/D RNP to these non‐natural substrates. Again, in a second reaction, the alkylated RNA molecules were fluorescently labeled by CuAAC.19a

Functionalization of both miRNA and small interfering RNA (siRNA) has been described by Plotnikova et al.19b Here, the HEN1 2′‐*O*‐methyltransferase from *Arabidopsis thaliana* was used to direct transalkylation to the 3′‐terminal nucleotides of small double‐stranded RNA molecules. In fact, this study demonstrates the direct transfer of biotin to the 3′ ends of small RNA duplexes and their subsequent isolation using streptavidin‐coated beads.19b

#### 2.4.3 Protein Transalkylation

The two most common types of AdoMet‐dependent protein MTases are protein arginine MTases and protein lysine MTases.^53^ However, there are some protein MTases that target other sites such as other peptidyl chains or the N or C termini of proteins.^54^ The main targets of arginine and lysine protein MTases are histones, for which methylation of the histone is associated with repression of transcription.^55^ Besides histones, numerous other proteins have also been targets of methylation, where they play a role in many physiological pathways such as signal transduction and protein translocation.^56^ Because of the role of histone methylation in the repression of transcription, protein MTases have been implicated in cancer, neurodegenerative diseases, and other diseases.^57^

One early study in 2001 demonstrated that a mutant of the yeast MTase *Rmt*1 was selectively inhibited by N^6^‐substituted AdoMet analogues. This was the first successful demonstration of the bump‐hole technique as a means to develop selective combinations of mutant methyltransferase enzymes and tailored AdoMet analogues.^44^ The aziridine‐based cofactor analogues and the PRMT1 protein methyltransferase have been successfully employed for protein transalkylation.^15^, ^58^ The doubly‐activated (mTAG) cofactors were first employed in protein transalkylation reactions by Peters et al.20e This and a later study describe the transfer of an alkyne group to the target of the histone H3K9 protein MTases Dim‐5 and SETDB1.^59^

The Luo group has developed combinations of several AdoMet analogues and engineered protein MTases to enable bioorthogonal targeting of transalkylation reactions in cells. Their work is covered in detail in a recently published review^7^ but we provide a brief overview here. The focus of their work has been the human protein MTases G9a (also known as EuHMT2), GLP1 (also known as EuHMT1), and PRMT1 (for a complete overview, see Table 2). Enzyme‐mediated transalkylation reactions using an azido‐modified AdoMet analogue with engineered G9a and GLP1^21^, ^60^ and an alkyne‐modified AdoMet analogue with engineered G9a20c and PRMT1 have been demonstrated.20g Additionally, a selenium‐based SAM analogue with an alkyne linker was effectively employed in transalkyation reactions directed and catalyzed by the native protein MTases GLP1, G9a and SUV39H2.^28^, ^60^, ^61^

**Table 2:** Protein MTases shown to be compatible with various AdoMet analogues and the functionalities implemented on the AdoMet analogue.

	MTAG			Aziridine		
	Alkyl	Alkyne	Azide	None	Alkyne	Azide
PRMT1^[a]^		20g,20h		15, 58	58	58
PRMT3		20a				
Dim‐5		20e,20h				
SETDB1^[a]^		59				
GLP1 (EuHMT1)^[a]^	20b	20b, 28, 62, 64	21			
G9a (EuHMT2)^[a]^	20b	20b,20c,20h, 28, 29, 62, 64	21,60			
METTL21A		29a				
METTL10		29a				
Set7/9		20h, 49d				
PrmC		20h				
SUV39H2		28, 62				

[a] Mutant enzyme with improved activity for the AdoMet analogue.

An extensive study with eight native enzymes showed limited transalkylation by the three wild‐type protein MTases G9a, GLP1, and SUV39H2 when using an allyl AdoMet analogue, and a complete absence of activity with the bulkier AdoMet analogues.^62^ However, a single mutation in these three enzymes was shown to have a dramatic impact on their ability to catalyze transalkylation reactions and, furthermore, led to a significant reduction in the rate at which they performed methylations using AdoMet. These mutants were thus found to be capable of performing transalkylation reactions with synthetic AdoMet analogues in the presence of native AdoMet (e.g., in cell lysates).^63^ In a subsequent study, Islam et al. investigated the function of the mutations Y1211A in EuHMT1 and Y1154A in EuHMT2, which further improved the enzymatic transalkylation rates when using the AdoMet analogues. The residues targeted for mutation were identified as gatekeeper amino acids that had blocked access to the cofactor binding pocket for the bulkier, synthetically prepared AdoMet analogues.20b

#### 2.4.4 Small‐Molecule Transalkylation

Small‐molecule or natural product methylation is ubiquitous across all branches of the tree of life. Many AdoMet‐dependent MTases that target natural products (NP MTases) exist and they can generally be categorized based on their preference for oxygen, nitrogen, sulfur, or carbon atoms as their nucleophilic substrates.^65^ As such, NP MTases participate in the modification of a large numbers of structurally diverse small organic molecules, which affects their bioavailability, activity, and reactivity in processes ranging from metabolism to signaling and biosynthesis.65b Many NP MTases have been shown to feature a Rossman‐like fold structural motif that is often extended by additional domains. These additions to the core enzyme structure ultimately allow individual NP MTases to display wide ranging substrate specificity. Because of their versatility, NP MTases, when used in combination with AdoMet or its artificial analogues, are particularly appealing in the context of biocatalysis and the production of fine chemicals, where they can help to unlock synthetic routes that would not be accessible using traditional methods. Excellent overviews of such efforts are provided elsewhere.2b, 66

## 3 Current and Future Applications

The ever expanding repertoire of doubly‐activated cofactor analogues and suitable natural or engineered MTases has provided us with a versatile set of tools for labeling the three major biopolymers.^67^ While their most immediate applications might be found in the study of the biological mechanisms underlying epigenetic modification and signaling, it would be hard to overstate the potential uses of such a highly specific biomolecular toolkit in all areas of research where directed, site‐specific labeling is required. In the following paragraphs, a selection of these applications is presented.

### 3.1 Selective Enrichment of Biomolecules

The specific enrichment of genetic material is particularly important in the context of high‐throughput, high‐coverage sequencing efforts.^68^ Indeed, even though next‐generation sequencing technologies have made whole‐genome sequence information relatively easy to obtain, the sheer amount of data produced can be a confounding factor. Moreover, diseases such as cancer or viral infections will result in sub populations of cells or even single cells with a distinct genetic makeup against the background of the entire population, giving rise to so‐called subclonal genetic heterogeneities.68b

By labeling target DNA, for example, with a biotin‐containing AdoMet analogue, it can subsequently be captured, for example by using streptavidin‐functionalized particles.19b Alternatively, biomolecules can be labeled using clickable AdoMet analogues and coupled to azide‐ or alkyne‐functionalized beads. This method was recently demonstrated using lambda DNA labeled with M.*Taq*I and a cofactor featuring a terminal alkyne.^69^ A different study showed DNA capture by labeling with either amine or azide moieties.^50^ Subsequently, the molecules were coupled to the surface of particles via either a biotin functionalized with an NHS ester adduct or biotin modified with a dibenzocyclooctyne moiety.

The use of MTases in the detection of non‐methylated genomic DNA has been demonstrated by the Klimašauskas group.^50^ The MTase M.*Sss*I was used to label DNA featuring regions of unmethylated CpG sites with a cofactor that could be used to biotinylate the DNA. This biotinylated DNA could subsequently be selectively captured for downstream analysis using microarray screening or sequencing.^50^

Next to the identification of genomic regions featuring low methylation, MTase‐based labeling strategies have also been applied to the separation of DNA of interest from a background of other genomic material in the sequencing of the Neanderthal genome. Here, environmental DNA of mostly bacterial origin needed to be removed. To achieve this, the researchers used the fact that mammalian DNA is frequently methylated at CpG sites, which occurs far less frequently in bacterial DNA. Treatment with restriction enzymes that specifically target CpG sites largely destroys the bacterial DNA, leaving the endogenous DNA intact.^70^ In the referenced application, Mtases are not directly used. Rather, the DNA of interest (the endogeneous DNA) is methylated in vivo by MTases whereas bacterial DNA is not. This allows separation of the two through simple restriction endonuclease treatment.

**Figure 3:**
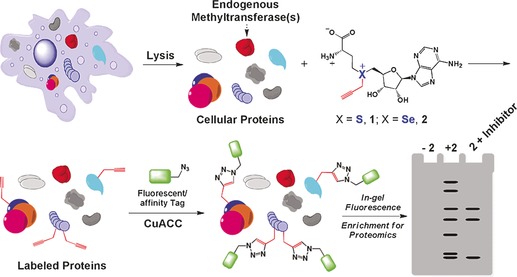
Use of an AdoMet analogue as a reporter of protein methylation. The AdoMet analogue can be utilized by endogenous methyltransferases to label cellular proteins with an alkyne moiety. The modified proteins can then be coupled to a fluorescent tag using CuAAC for further characterization. Reproduced from Ref. 21.

Capturing biomolecules is not just limited to DNA, but can potentially also be used to specifically enrich RNA or proteins. Indeed, it has been shown that proteins can be captured by coupling a biotin tag to proteins with click chemistry.^71^ This approach has been used by the Luo group for the specific targeted capturing of proteins using MTase‐based labeling (Figure 3).20a,20b, 28, 64

Specifically, an AdoMet analogue with alkyne functionality was used for targeted labeling of protein MTase targets. Then, biotin modifed with an azide group was coupled to the proteins using click chemistry, and the proteins were subsequently captured using streptavidin beads. The presence of a cleavable azo linker between the azide and biotin made it possible to separate the captured proteins from the beads after treatment with sodium dithionite (Na_2_S_2_O_4_). Finally, the strategies presented here might also be useful in the assembly of biomolecular structures49b and the positioning of nanoparticles.49a

### 3.2 Genomic Analysis

#### 3.2.1 DNA Mapping

The discovery of sequence‐specific restriction endonucleases (REases) enabled DNA sequence analysis long before the advent of single‐nucleotide sequencing.^72^ When a DNA sample is digested by a known panel of REases, the ensuing characteristic fragment lengths can be analyzed using agarose gel electrophoresis, which results in a “DNA fingerprint” with applications in forensics, for example,.^73^ Schwartz et al. further refined the technique by binding linearized DNA molecules to a surface prior to digestion, which results in restriction or sequence “maps” where additional information is contained in the relative location of the different fragments.^74^ Site‐specific incorporation of fluorescent labels, rather than actual restriction of the DNA, has further contributed to increase the information content of sequence maps.^75^ Whereas nicking endonucleases and corresponding fluorescent nucleotide analogues were initially used here, they have since been supplanted by MTase‐mediated labeling, which offers a number of advantages: 1) The use of MTases keeps the DNA backbone intact, thereby improving the stability of the DNA molecules and minimizing unwanted fragmentation. 2) DNA MTases enable a more direct labeling approach compared to nicking endonucleases, which increases the efficiency of transfer. 3) Through careful choice of the DNA MTases, high densities of labeling can be achieved. Indeed, a DNA MTase with a 4‐base recognition sequence applied to a random DNA sequence would result in 1 label every 256 base pairs (4^4^).

**Figure 4:**
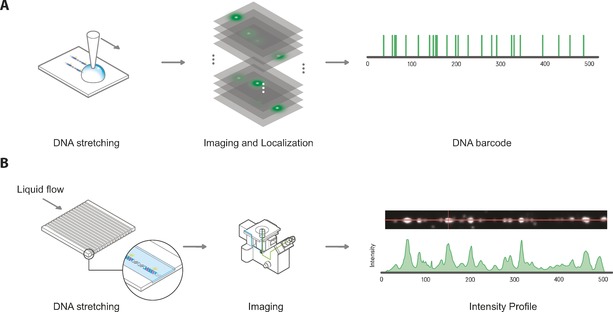
The two main methods for reading the distribution of fluorophores on DNA molecules. A) For high‐resolution localization of fluorophores on the DNA, the DNA needs to be rigidly attached to the surface. B) For rapid imaging of DNA, the DNA molecules can be passed through nanochannels where they are elongated and subsequently, the intensity pattern can be recorded.

The past couple of years, several approaches to DNA mapping using DNA MTases have been developed, and excellent reviews on the subject exist.^76^ These approaches can be roughly categorized in DNA mapping in nanofluidic channels and DNA mapping using super‐resolution microscopy (Figure 4).

MTase mapping in nanofluidic channels was first demonstrated by the Ebenstein group.^23^ Here, phage T7 and phage Lambda DNA were labeled using the methyltransferase M.*Taq*I (recognition sequence: 5′‐TCGA‐3′) and a synthetic AdoMet analogue carrying the fluorophore TAMRA. The labeled molecules were subsequently stretched in nanochannels, after which intensity profiles were extracted using a fluorescence microscope. Because of the high density of fluorophores in combination with the use of diffraction‐limited microscopy, the exact location of the labels could not be determined. Instead, the obtained intensity profiles were matched to several computationally generated intensity maps through cross‐correlation of the profiles. Thresholding the cross‐correlation scores allows efficient matching of bacteriophages to the correct sequence. More recently, the same group also demonstrated the ability to perform genomic mapping with sub‐diffraction‐limit resolution in silicon nanochannels, thereby greatly enhancing the information density.^77^

Due to the high density of labels, the work in our laboratory has focused on extracting high‐resolution localization information from the DNA molecules.19c, 20f To be able to extract high‐resolution localization information from the DNA, it is important that the molecules are fixed on the surface. One method for stretching DNA molecules on a surface is based on flow stretching and attaching the stretched DNA to a surface coated with poly‐l‐lysine. In one such example, bacteriophage T7 (40 kbp) labeled with M.*Bse*CI (recognition sequence: 5′‐ATCGAT‐3′) and a biotin‐containing aziridine‐modified biotin analogue was coupled to streptavadin‐coated quantum dots. The DNA molecules were subsequently stretched on the surface using capillary flow.49c Due to the sparse labeling, the labels could be easily localized. However, the localization suggests there is a large variation in the positioning owing to deposition inhomogeneity. An alternative method for stretching DNA is based on DNA combing, a method developed by Bensimon et al. in the 90s.^78^ With this approach, DNA transalkylation using AdoMet analogues carrying either terminal amine or alkyne groups was carried out with the M.*Hha*I and M.*Taq*I (recognition sequences 5′‐TCGA‐3′ and 5′‐GCGC‐3′) methyltransferases. This gave a high density of modified sites, which were subsequently labelled using NHS ester or azide derivatives of the Atto647N dye. Following deposition, sub‐diffraction‐limit imaging was achieved based on stochastic photobleaching and localization of individual emitters.76a This resulted in an approximate resolution of 42 nm (approximately 80 base pairs).19c

Both these methods suggest a promising new technique for extracting sequence information from DNA molecules by studying the position of MTase‐mediated labeling on DNA. One example is the typing of bacteriophage DNA molecules based on a barcode embedded in the molecule. Another application could be the detection of copy‐number variation, repeats of large genomic elements in the genomes that are hard to find by sequencing.76a Finally, the MTase‐based labeling approach can easily be combined with other genomic analysis approaches such as fluorescence in situ hybridization (FISH).

#### 3.2.2 DNA Localization and Spatially Resolved Transcriptomics

The attachment of reporter groups to biomolecules allows their localization in situ. This has enabled researchers to put detailed functional analysis of biological processes in a spatial context, thereby allowing biological function, that is, molecular genetics and biochemistry, to be correlated with information on biological structure (obtained from embryology and histology, for example).^79^

Fluorescent labeling of nucleic acids such as DNA is one of the easiest routes to their localization in cells, and many methods in fact exist.^**[80]**^ However, MTase‐mediated transfer of fluorescent groups offers the particular advantage that it allows controlled targeting of the label and its covalent attachment.

In an example from the Weinhold group, Cy3 dyes were covalently coupled to pUC19 and pBR322 plasmids using an N‐adenosylaziridine cofactor.^51^ In this study, the labeled plasmids were successfully transfected into CHO‐k1 cells. Of these transfected cells, 25 % of the cells showed a high Cy3 fluorescence intensity in the nucleus, despite the absence of a nuclear import sequence on the plasmids.^51^

**Figure 5:**
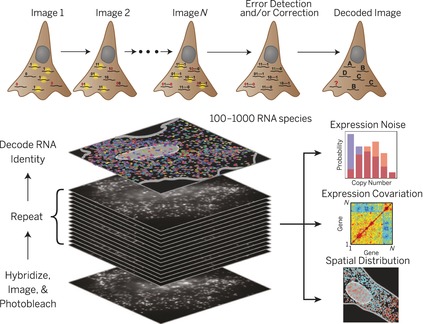
In the highly multiplexed RNA profiling method of the Zhuang group^81^ hundreds of individual mRNA can be correctly be identified and localized in situ.

It can be envisioned that MTase‐based labeling strategies could equally contribute to facilitate the in situ study of large scale regulatory networks or efforts in highly multiplexed transcription profiling, as recently demonstrated Zhuang and co‐workers (Figure 5).^81^ Here, the authors used an elaborate library of hybridization probes that were fluorescently labeled and designed to target specific RNA species. By designing multiple, differently labeled probes, and using multiple rounds of hybridization, the authors were able to impart a unique color coding to tens to even hundreds of individual RNA species in situ (Figure 5).

Although this example constitutes a great technical and scientific feat, the requirement to design and subsequently use hybridization probes can prove challenging, and indeed, the authors had to apply significant error correction concepts borrowed from computer data encoding to their design of the probes. Furthermore, although this study allowed the simultaneous tracking of large numbers of biomolecular species, it was still limited to RNA. Therefore, the possibility of achieving of highly directed MTase‐mediated labeling of all major classes of biomolecules opens up enticing new prospects to apply massively parallel observation of these species in an effort to truly unravel complex, spatially organized regulatory networks and elucidate cell‐to‐cell variations in the context of whole tissues.^79^

### 3.3 Epigenetic Analysis

Epigenetic regulation is a collective term used to denote the entire spectrum of processes that modulate gene activity in an organism without actually altering the genetic sequence. Cytosine methylation (5mC) and stepwise conversion of the 5mC into hydroxymethyl‐ (5hmC), formyl‐ (5fC) and, finally carboxyl‐ (5caC) cytosine through TET‐enzyme‐mediated demethylation are common in mammalian cells.^82^ These modifications play important roles in embryonic development, stem‐cell differentiation, genomic imprinting, neuronal function, and cancer.^83^ Additionally, histones and transcription factors can also be modified through methylation, acylation, or phosphorylation.^84^ Because of their role in methylation, MTases, together with suitable AdoMet analogues, are the ideal tools to study these diverse and transient phenomena.

#### 3.3.1 DNA Methylation

Bisulfite conversion is a commonly used method for the detection of 5mC, as well as the other cytosine modifications. It relies on chemical modification of the target species followed by PCR or sequencing‐based quantification.^83^, ^85^ Unfortunately, the method suffers from relatively low sensitivity, particularly for low‐abundance modifications, as well as relatively high error rates.^83^ Furthermore, the method only provides ensemble‐averaged information, whereas the stochastic nature of DNA methylation calls for approaches with single‐cell resolution.^86^ In this context, single‐molecule detection methods can offer a solution. In one example, the methylation status of single DNA molecules was probed using restriction enzymes for which activity is blocked by the methylated target base. Combining this with optical mapping results in a methylation map of the DNA.^87^ More recently, methyl‐CpG‐binding proteins were used on DNA stretched in nanochannels,^88^ thereby allowing direct detection of the methylation status of single molecules. A more direct method was used for the detection of hydroxymethylcytosine. Here, the enzyme T4 β‐glucosyltransferase was used to attach glucose molecules with a reactive azide moiety to the hydroxymethyl group, which could subsequently be coupled with fluorescent dyes.^89^ For a more complete overview of the field, the reader is referred to several excellent reviews.^76^

**Figure 6:**
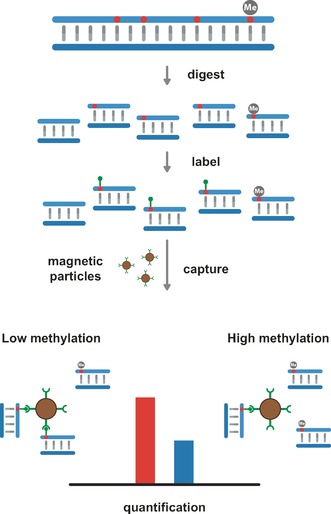
General principle for capture‐based detection of the unmethylated part of the genome, illustrated with a quantitative PCR assay.

Like restriction enzymes, a methylated base also blocks the activity of methyltransferases. This characteristic was explored in a recent paper that demonstrated profiling of the unmethylated part of the genome (the “unmethylome”).^50^ Unmethylated sites were labeled with the CpG MTase M.*Sss*I and an AdoMet analogue containing either an azide or amine functionality. The labeled DNA was then coupled to biotin (biotin functionalized with DBCO or an NHS ester) and extracted using streptavidin‐coated microbeads. After purification, the DNA was analyzed using microarrays (Figure 6). This approach suggests a relatively straightforward way of enriching and subsequently sequencing unmethylated DNA. Because of the sensitivity of the MTase‐based method, very low amounts of DNA (100–300 ng) are needed to complete the analysis. Furthermore, the method was particularly sensitive to regions in the genome with low CpG presence, something that presents difficulties for other enrichment techniques such as methylated DNA immuneoprecipitation (MeDIP) and methyl‐CpG binding domain capture (MBD).^50^

MTase‐based labeling of methylated DNA could easily be combined with single‐molecule detection in nanochannels.76b Since MTase‐based labeling can be targeted towards either adenine or cytosine, the method could be combined with optical mapping in order to localize regions of (un)methylation. Epigenome mapping could then be performed with a dual‐color approach, with one color for a map of the DNA, and another color to create a map of the (un)methylated regions.

#### 3.3.2 Protein Methylation

The target substrates of protein methyltransferases have been extensively studied by the Luo group through the development of an assay termed bioorthogonal profiling of protein methylation (BPPM). Cells are transfected with a mutated protein MTase that shows a reduced preference for natural AdoMet compared to the analogue. Upon lysis, the mixture is incubated with a doubly‐activated AdoMet analogue with an azide linker. Target proteins are then coupled to a clickable dye, for example, dibenzylcyclooctyne‐coupled dyes, and identified by mass spectrometry (MS) analysis or sodium dodecyl sulfate polyacrylamide gel electrophoresis (SDS‐PAGE; Figure 3).^21^, ^60^, ^61^ The approach was used for detecting the substrates of the human protein MTase PRMT3, which preferably resides within the cytoplasm.20a Interestingly, while the protein MTase localizes in the cytoplasm, 23 % of the modifications were found in the nucleus, thus suggesting a broader role for PRMT3.

More recently, Luo et al. set out to study the methylation status of histones in live cells by hijacking the enzymatic synthesis of AdoMet to create AdoMet analogues instead. A modified version of methionine carrying a clickable group (a terminal alkyne) was transported into the cells and, together with ATP, processed by an engineered MAT to form the modified cofactor. This modified cofactor was then used with a mutant of G9a, which successfully transferred the alkyne group to the histones. The transfer of the alkyne group to histones was subsequently confirmed using LC–MS.^64^ The authors subsequently used this method to attach clickable (azide‐conjugated) biotin to the labeled chromatin. This allowed them to enrich the labeled chromatin using streptavidin‐coated beads. After cleavage of the linker, the DNA attached to the histones could be purified and sent for sequencing.^64^ Indeed, qPCR analysis confirmed the presence of several genes that reside on the targeted histones. Because of the structural similarity between different MTases, this method is expected to be applicable across a wide array of protein MTases with varying targets. For more details on the detection and analysis of protein methylation, the reader is referred to one of several reviews on the topic.^90^

## 4 Conclusions and Outlook

Since the conception of labeling biomolecules using MTases, much work has been done. The method offers a simple solution for placing reporter molecules specifically and covalently onto target biomolecules. So far, the method has been convincingly shown with more than 20 MTases: 9 DNA MTases, including both adenine and cytosine DNA MTases, 4 RNA MTases, and 11 protein MTases. Several mutated versions of DNA or protein MTases have been developed with improved activity for the various AdoMet analogues, and in some cases a preference of the MTase for the AdoMet analogue over the natural cofactor AdoMet. This preference allows the method to be used in in vivo systems where the natural cofactor AdoMet is still present in the sample mixture.

Two major groups of AdoMet analogues are currently available for MTase‐based labeling. The aziridine‐based cofactors are synthesized chemically and can be used in MTase‐mediated labeling reactions in which the whole compound is transferred to the substrate. The largest group of AdoMet analogues are the double‐activated AdoMet analogues, in which the transferrable methyl group of AdoMet is replaced by an unsaturated alkyl group that is used in mTag labeling of biomolecules. These analogues are typically synthesized from the precursor AdoHcy. Interestingly, these AdoMet analogues can sometimes also be synthesized by hijacking the same enzymes that create the natural cofactor AdoMet in cells, thereby greatly simplifying the synthesis.

This approach for the targeted labeling of biomolecules can subsequently be used for all sorts of applications. The attachment of biotin or other chemical groups makes it possible to specifically capture low quantities of DNA, mRNA, or proteins. Furthermore, the method offers a way to covalently attach organic fluorescent molecules to target molecules, which makes it useful for detection using microscopy‐based methods. Finally, the targeted labeling of DNA offers a way to analyze sequence information beyond the standard sequence reads, either by imaging far longer DNA molecules or by focusing on the epigenetic code embedded in the DNA.

The development of methyltransferases as tools for biotechnology is a story that showcases the impact that basic chemistry can have in this field. The emerging applications we have discussed highlight the potential of MTase‐mediated reactions as a general strategy for targeting and labelling specific sites complex biological samples. There remains significant work to be done to bring these tools to the non‐specialist laboratory, but the promise we have outlined should prove a strong driver for future development.

## Conflict of interest

J.H., R.K.N. and V.L. have founded a company that may market applications of artificial AdoMet analogues for biofunctionalization.

## Biographical Information

*Johan Hofkens received his M.Sc. and Ph.D. degrees in Chemistry from KU Leuven under supervision of Prof. Frans C. De Schryver. After postdoctoral research with Prof. Prof. Hiroshi Masuhara at Osaka University and Prof. Paul Barbara at the University of Minneapolis, he rejoined KU Leuven, where he started the Single Molecule Unit. In 2005 he was appointed Research Professor at KU Leuven, and in 2008 he was promoted to full professor. His research interests are single‐molecule spectroscopy, fluorescence and non‐linear microscopy, and the application of these techniques in materials science and biosciences, for which he received an ERC advanced grant in 2012*.

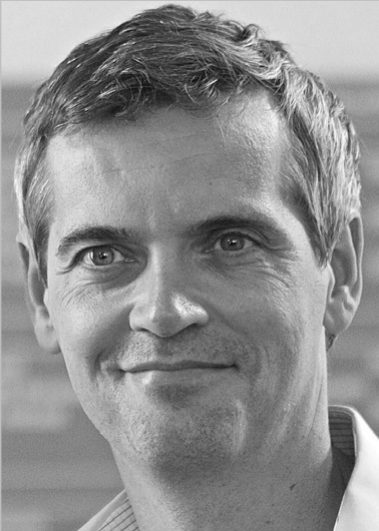

## Biographical Information

*Kris Janssen obtained an M.Sc. degree in Bioscience Engineering and Catalysis in 2005 from KU Leuven. After fulfilling positions in private research and industry, he returned to KU Leuven in 2008 to pursue a Ph.D. degree in the group of Prof. Jeroen Lammertyn, focusing on the development of DNA‐based biosensors. After graduating in 2013, he took up a position as an FWO postdoctoral fellow and is now working with the group of Prof. Johan Hofkens. His research interests lie in the application of super‐resolution fluorescence and electron microscopy to study the interactions of DNA, RNA, and proteins with inorganic nanomaterials*.

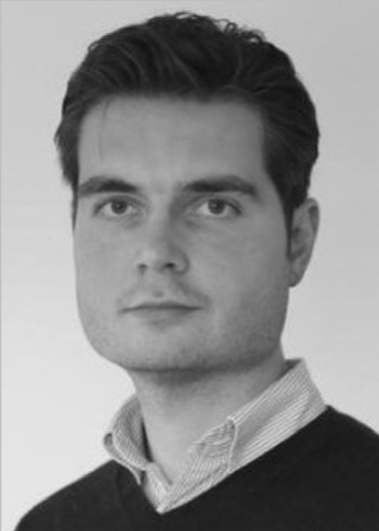

## Biographical Information

*Jochem Deen received his B.Sc. in engineering from Fontys Eindhoven in 2008 and a M.Sc. in biophysics in 2010 from KU Leuven. He obtained his Ph.D. in the group of Prof. Johan Hofkens in 2016 with Prof. Rob Neely as a cosupervisor. During his Ph.D., he focused on the development of DNA mapping using DNA methyltransferases and super‐resolution fluorescence microscopes*.

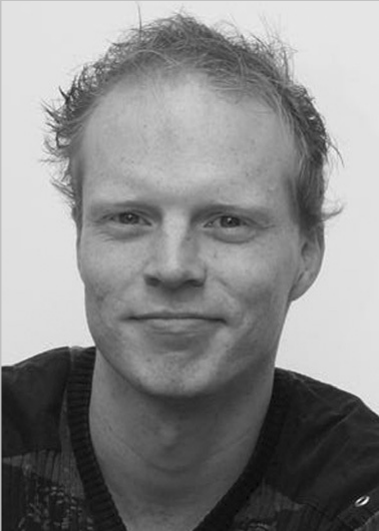

## Biographical Information

*Charlotte Vranken obtained an M.Sc. degree in Chemistry at KU Leuven in 2011. She obtained her Ph.D. under the supervision of Prof. Johan Hofkens and Wim Dehaen in 2016. Her research was focused on the synthesis of AdoMet analogues for sequence‐specific DNA modification*.

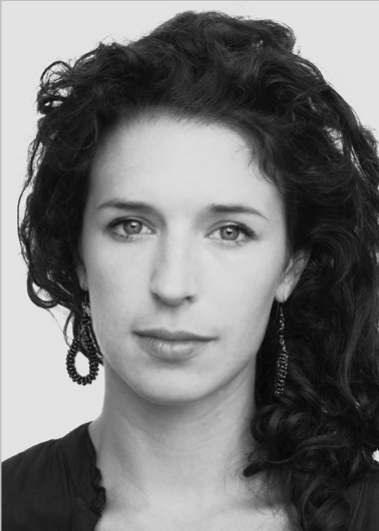

## Biographical Information

*Rob Neely is currently a Senior Lecturer and EPSRC Healthcare Technologies Fellow at the University of Birmingham. He obtained his Ph.D. in 2005 with Prof Anita Jones at the University of Edinburgh. He was awarded an EPSRC postdoctoral fellowship (Edinburgh) and spent a year with Sir Richard Roberts at New England Biolabs (Massachussets), working on DNA methyltransferases. He moved to Prof. Hofkens’ group at KU Leuven Belgium in 2009 as a Marie Curie Fellow and was appointed Senior Lecturer in Birmingham in 2014*.

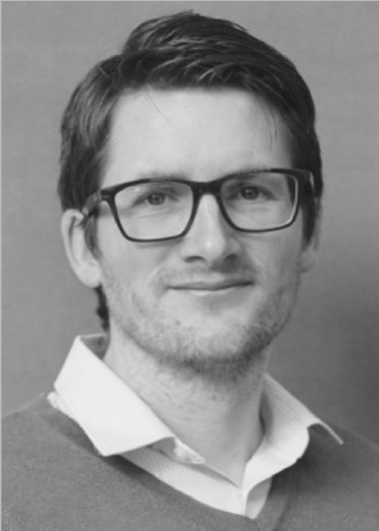

## Biographical Information

*Volker Leen obtained M.Sc. and Ph.D. degrees in Chemistry (Organic Synthesis) from KU Leuven under supervision of Prof. Wim Dehaen in 2010, focusing on pyrrole and dye synthesis. After postdoctoral studies on antiviral compounds in the group of Prof. Pof. Wim Dehaen, he joined the research Group of Johan Hofkens. His current research interest is biotechnology development from an organic synthesis point of view*.

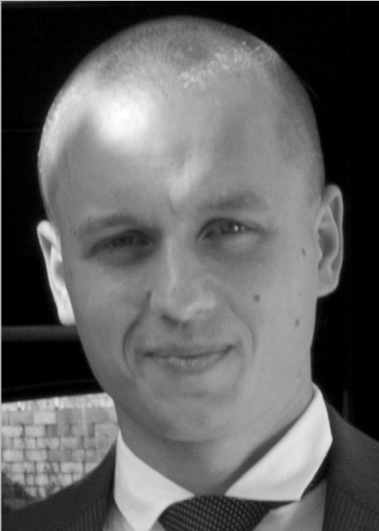

## Supporting information

As a service to our authors and readers, this journal provides supporting information supplied by the authors. Such materials are peer reviewed and may be re‐organized for online delivery, but are not copy‐edited or typeset. Technical support issues arising from supporting information (other than missing files) should be addressed to the authors.

Supplementary
