# Supplementary material for: Methyltransferase‐Directed Labeling of Biomolecules and its Applications
Source: Angew Chem Int Ed Engl. 2017 Apr 10;56(19):5182–200. doi: 10.1002/anie.201608625 (PMC5502580; doi:10.1002/anie.201608625)
Supplement: Supplementary file 1 — Supplementary [file ANIE-56-5182-s001.pdf]

Angewandte Chemie
Eine Zeitschrift der Gesellschaft Deutscher Chemiker

Supporting Information

Methyltransferase-Directed Labeling of Biomolecules and its Applications

Jochem Deen+, Charlotte Vranken+, Volker Leen, Robert K. Neely, Kris P. F. Janssen, and Johan Hofkens**

anie_201608625_sm_miscellaneous_information.pdf

Supplementary information

Table 1: Aziridine based AdoMet analogues

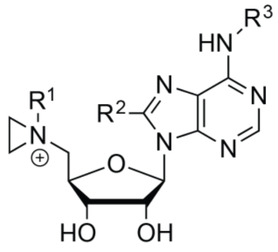

Aziridine based
AdoMet analogue

Compounds	R^1	R^2	R^3
**12**	H	N_3	H
**13**	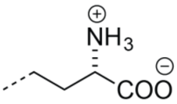	H	H
**14**	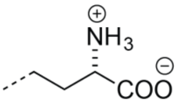	N_3	H
**15**	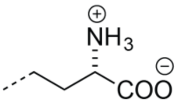	$---NH(CH_2)_4N_3$	H
**16**	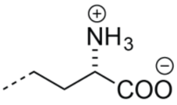	H	$---CH_2C{\equiv}CH$
**17**	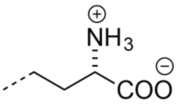	$---NHCH_2C{\equiv}CH$	H
**18**	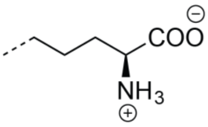	H	H

Table 2: Doubly activated AdoMet analogues

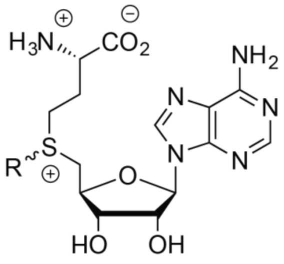

Doubly activated
AdoMet analogue

Compounds	R	Compounds	R
**19**	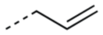	**25**	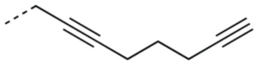
**20**	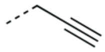	**26**	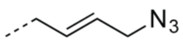
**21**	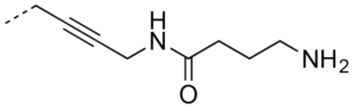	**27**	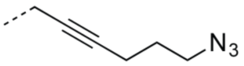
**22**	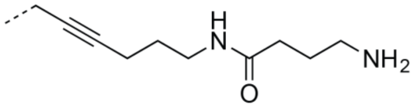	**28**	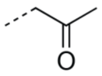
**23**	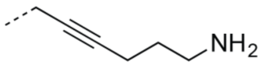	**29**	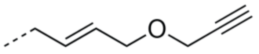
**24**	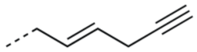		
